# Combined Effects of Sulfamethoxazole and Erythromycin on a Freshwater Microalga, *Raphidocelis subcapitata*: Toxicity and Oxidative Stress

**DOI:** 10.3390/antibiotics10050576

**Published:** 2021-05-13

**Authors:** Yibo Zhang, Da He, Fang Chang, Chenyuan Dang, Jie Fu

**Affiliations:** 1 School of Environmental Science and Engineering, Huazhong University of Science and Technology, Wuhan 430074, China; ybo_zhang@sina.com (Y.Z.); jiefu@hust.edu.cn (J.F.); 2 Key Laboratory of Ecological Impacts of Hydraulic Projects and Restoration of Aquatic Ecosystem of Ministry of Water Resources, Institute of Hydroecology, Ministry of Water Resources and Chinese Academy of Sciences, Wuhan 430079, China; dahe@mail.ihe.ac.cn; 3 Marine Resources Research Centre, Tianjin Research Institute for Water Transport Engineering, M.O.T., Tianjin 300456, China; changfang1999@163.com

**Keywords:** microalgae, sulfamethoxazole, erythromycin, toxicity, antioxidant system

## Abstract

This study investigated the environmental effects of two familiar emerging contaminants, sulfamethoxazole (SMX) and erythromycin (ERY), and their mixture (10:1 *w*/*w*) using a green microalga, *R. subcapitata*. The cell density, pigment content, and the activities of superoxide dismutase (SOD), catalase (CAT), glutathione (GSH) glutathione peroxidase (GSH-Px), and glutathione S-transferase (GST) were analyzed. The calculated EC_50_ values of SMX, ERY, and their mixture after 96 h were 0.49, 0.044, and 0.06 mg/L, respectively. High concentrations of antibiotics lead to a decrease in chlorophyll a and total carotenoid content, affecting the ability to photosynthesize ROS scavenging capacity. This may be a factor leading to the inhibition of algal growth. When *R. subcapitata* was exposed to SMX and the mixture, SOD and CAT increased to resist oxidative damage, while the activities of GSH and GST decreased, suggesting that this algae’s antioxidant system was unbalanced due to oxidative stress. *R. subcapitata* reduced the ERY-induced ROS by increasing the activities of SOD, GSH, and GST. The difference in the contents of nonenzymatic antioxidants and enzyme antioxidants in *R. subcapitata* indicated the antioxidant mechanisms to SMX and ERY were not identical. This study provides insights into the oxidative stress process in *R. subcapitata* under different antibiotics.

## 1. Introduction

Pharmaceuticals and personal care products (PPCPs) are over consumed daily and are transported to the aquatic environment. PPCPs are considered a type of emerging organic contaminant (EOC), which cover various groups of organic chemicals (such as antibiotics, hormones, and musk fragrances) and have received surprising concern in recent years. Thousands of tons of antibiotics are produced annually worldwide and are widely used in medicine and animal husbandry [1]. Antibiotics are released into the environment through several ways such as human excretion, improper disposal, and discharge from manufacturing sites and then reach wastewater treatment plants (WWTPs). Therefore, WWTPs are the predominant way of human pharmaceutical discharge into the aquatic environment [2]. Although most antibiotics are not highly persistent, their consecutive production and discharge still lead to pseudo-persistent contamination and come into play through the interaction of specific pathways in target organisms at low concentrations [3,4]. Nontarget organism exposure to antibiotics gives rise to adverse effects. The determination of toxicity to nontarget organisms is beneficial to comprehend the adverse impact on ecosystems.

Algae as primary producers in the food chain play a pivotal role in maintaining the function of ecosystems [5]. Therefore, algae have been proposed as a pivotal indicators for evaluating the water quality and ecotoxicity of chemicals [6]. Although the target organism of antibiotics is bacteria, antibiotics may still have adverse effects on algae, especially for chloroplasts, mitochondria, and other organelles of algae [6]. When algae are exposed to exogenous pollutants, it is possible to accumulate reactive oxygen species (ROS) in algae cells [7]. In order to prevent ROS from causing fatal damage to cells, specific nonenzymatic and enzymatic antioxidants are produced in the cell to eliminate ROS production. Green algae (*Raphidocelis subcapitata*) are a model species commonly used in chemical tests (OECD 2011) [8]. However, there are different suppressive responses in algae when they are exposed to different antibiotics. As things stand at present, an integrated evaluation of the tolerance mechanisms of microalgae is not well established. In addition, mixed pollution of different antibiotics is a real problem in water bodies. The integrated effects of these pollutants should also be completely studied when assessing their environmental risk.

Recent studies on antibiotics have focused on sources, fate, and removal. However, the potential effects on algae have not drawn much attention [9]. Previous studies have shown that antibiotics have a certain effect on the growth of algae, including inhibition and hormesis [9,10]. Most studies were performed at high concentrations in short-term exposure (72/96 h) to investigate acute toxicity. However, the persistence of antibiotics in the aquatic environment, meaning the response of algae to the long-term exposure of antibiotics, is practically significant. Considering that algae may show different responses to external environmental pressures, we observed the responses of algae to two common antibiotics of SMX and ERY in simulated natural conditions. In this study, we focused on algae that received long-term exposure to the single and combined effects of the antibiotic mixture. In addition, we also studied the response of the antioxidant system and pigments, which may further regulate algal growth and numerous physiological processes. Therefore, the goal of this study is to evaluate the toxicity of SMX, ERY, and their mixture on *R. subcapitata* according to the growth patterns and to investigate the effects of SMX, ERY, and their mixture on antioxidant responses and pigments.

## 2. Results and Discussion

### 2.1. Toxicity of SMZ, SMX, and Their Mixture

**Figure 1:**
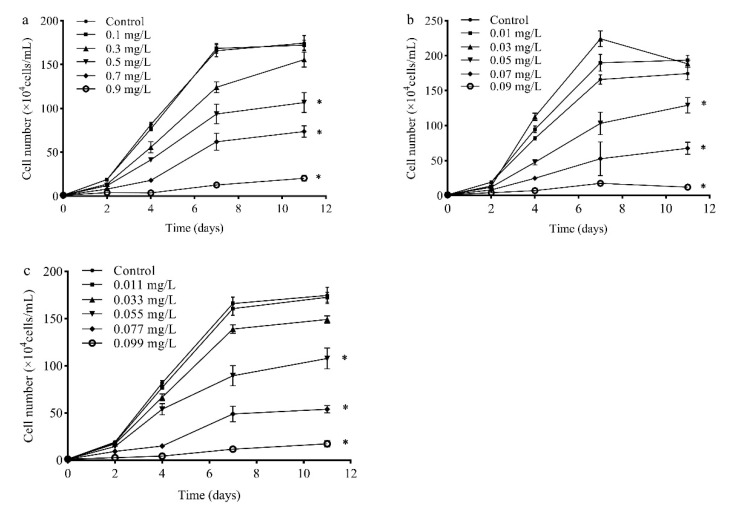
Effects of (**a**) SMX, (**b**) ERY, and (**c**) their mixture on the growth of *R. subcapitata* in different concentrations. Data are presented as mean values ± standard deviation (n = 3). The asterisk (*) indicates a significant difference (*p* < 0.05) between control and treatments.

Microalgae have been recommended as a model organism for toxicity evaluation by the OECD [5]. The algae cells of *R. subcapitata* varied following treatment with SMX, ERY, and their mixture at different concentrations during 11-day test periods. In the present work, *R. subcapitata* had negligible influence by SMX (≤0.3 mg/L) during all test periods. The harmful effects of SMX on *R. subcapitata* increased at concentrations of 0.5–0.9 mg/L. Compared with other test times, the highest influence was observed as 6.4–95.0% growth inhibition during 4 days of cultivation, respectively. After 4 days, *R. subcapitata* might develop adaptations, thus the inhibition effect of SMX on *R. subcapitata* was weakened (Figure 1a). ERY (0.05–0.09 mg/L) showed an inhibition rate of 25.9–93.1%, as compared to the control. However, ERY (≤0.03 mg/L) produced hormesis on *R. subcapitata* (Figure 1b). In the case of the mixture, the concentration was greater than 0.055 mg/L, and the algae were significantly inhibited. The inhibition of *R. subcapitata* growth decreased after 4 days (Figure 1c).

Because antibiotics are usually present as mixtures in the environment, their combined effects are important to consider [11]. Combined mixtures can be nonadditive (synergism and antagonism). The half-maximal effective concentration (EC_50_) values of SMX, ERY, and their mixture were 0.49, 0.044, and 0.06 mg/L at 96 h, respectively. It is clear that the combination of low concentrations of SMX and ERY is more toxic than the toxic combination alone. EU Directive 93/67/EEC classified chemical substances on the basis of their EC_50_ against aquatic organisms into different categories: harmful (10–100 mg/L), toxic (1–10 mg/L), and very toxic (<1 mg/L). In the present study, our study data indicated that SMX, ERY, and their mixture can be classified as very toxic. Previous studies have investigated the effects of SMX and ERY on the growth of different algal species, whereas an evident difference in species sensitivity was detected (Table 1).

The difference in EC_50_ values could be explained by the different sensitivity of various microalgal species [15]. EC_50_ values gradually grew larger between 4 and 11 days, indicating that *R. subcapitata* develop adaptive mechanisms and/or the tested antibiotics are transformed into small molecules of low toxicity [16]. A couple of studies have investigated the effects of SMX and ERY on the growth of other algal species, whereas an evident difference in species sensitivity was detected (Table 2). The distinction in EC_50_ values may be due to the diverse sensitivity of different algae species. Furthermore, the RQs of SMX, ERY, and their mixture were 8.31, 7.95, and 73.67 in surface water. In general, the concentrations of antibiotics are higher in wastewater than in surface water. The RQs of SMX, ERY, and their mixture were 23.22, 18.86, and 211.83 in wastewater. SMX, ERY, and their mixture pose appreciably ecological risks to the aquatic environment.

### 2.2. Effects of Antibiotics on Pigments

**Figure 2:**
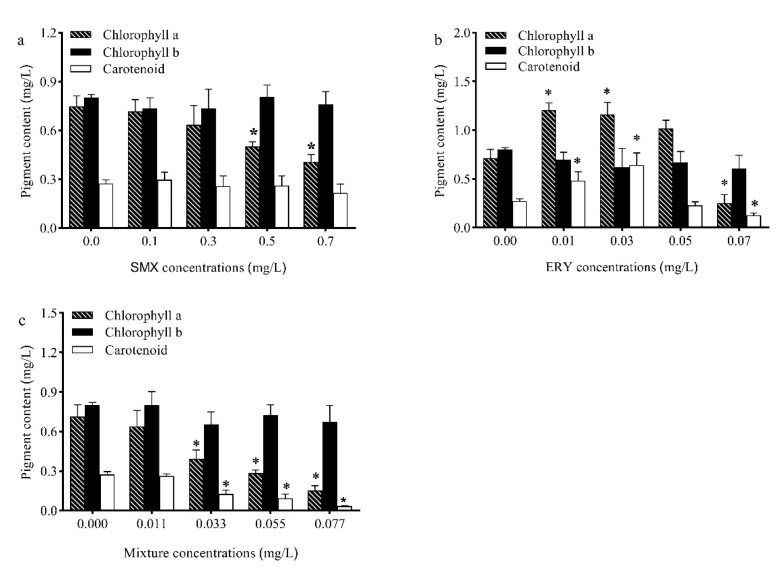
Effects of (**a**) SMX, (**b**) ERY, and (**c**) their mixture on the total chlorophyll and carotenoid contents of *R. subcapitata*. Data are presented as mean values ± standard deviation (n = 3). The asterisk (*) indicates a significant difference (*p* < 0.05) between control and treatments.

Chlorophyll is a pigment contained in higher plants and all other organisms capable of photosynthesis. It is closely involved in all stages of photosynthesis, including light harvesting, energy transfer, and light energy conversion. Therefore, changes in the growth of microalgae when exposed to toxic compounds are always related to chlorophyll biosynthesis [7]. On day 11, the contents of chlorophyll a exposed to SMX and the mixture were decreased significantly in *R. subcapitata* at a relatively high concentration (Figure 2a), while the contents of chlorophyll a exposed to ERY were increased significantly at low concentrations and decreased significantly at high concentrations (Figure 2b). In the present research, the contents of chlorophyll b had no significant change in all tests (Figure 2a–c). The increase in chlorophyll content is a self-protection mechanism of cells, which removes the accumulated ROS in chloroplasts. The increased total chlorophyll content of *C. mexicana* and *M. resseri* aeruginosa was evident when exposed to enrofloxacin [15]. A reduction in photosynthetic pigments is also a common stress response in plants and microalgae and can be caused by the decreased biosynthesis and/or increased degradation of chlorophyll, both resulting in decreases in photosynthetic rates [22]. It is widely accepted that chlorophyll degradation involves hydroxyl radicals produced by reactions between superoxide anion and H_2_O_2_ [23]. Photosynthesis provides enough energy for algae growth and cell division. Chlorophyll is extremely crucial for photosynthesis, as a decrease in the chlorophyll content can be problematic for the algae [24]. 

Carotenoids have antioxidant characteristics and maintain cells against free radicals, inhibit lipid peroxidation, and increase the stability of the photosynthetic apparatus and protection of integrity membranes [25]. The carotenoid content of *R. subcapitata* significantly increased under ERY (0.01 and 0.03 mg/L) stress then decreased under high ERY concentrations (Figure 2b), while the contents of carotenoid exposed to SMX had no significant change (Figure 2a). However, the contents of carotenoids exposed to the mixture had a significant change at high concentrations (Figure 2c). Previous studies have shown that microalgae can boost carotenoid content when they are exposed to selenium stress, nutrient deficiencies, and hormone treatment [26,27]. Carotenoids have the ability to protect algae cells from oxidative stress induced by pollutants [28]. Carotenoids can deactivate excited chlorophyll to prevent the stress-induced damage of the photosynthetic system, caused by the formation of ROS under high irradiation, low temperature, salinity, and exposure to toxic pollutants [29].

### 2.3. Effects of Antibiotics on Antioxidant Responses

**Figure 3:**
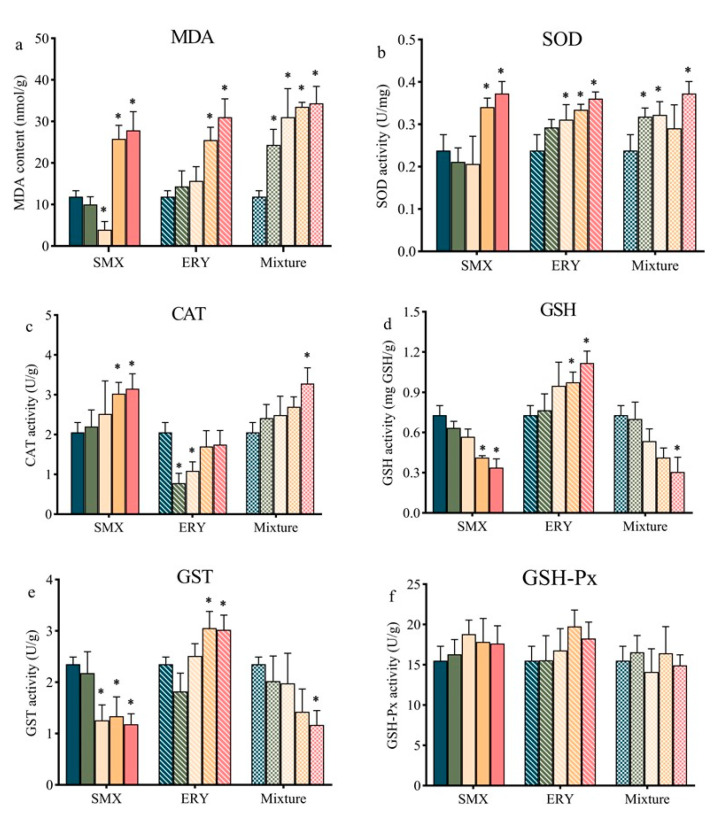
Contents of (**a**) malondialdehyde (MDA) and activities of (**b**) superoxide dismutase (SOD), (**c**) catalase (CAT), (**d**) glutathione (GSH), (**e**) glutathione S-transferase (GST), and (**f**) glutathione peroxidase (GSH-Px) in *R. subcapitata* after 11 days exposure to SMX, ERY, and their mixture. SMX (0, 0.1, 0.3, 0.5, and 0.7 mg/L), ERY (0, 0.01, 0.03, 0.05, and 0.07 mg/L), and their mixture (0, 0.011, 0.033, 0.055 and 0.077 mg/L in a ratio of SMX/ERY = 10:1). Data are presented as mean values ± standard deviation (n = 3). The asterisk (*) indicates a significant difference (*p* < 0.05) between control and treatments.

Under a physiological state, the level of cellular ROS is stable in a dynamic equilibrium, and this balance is modulated by cellular processes that produce ROS and eliminate them [30]. However, under exposure to antibiotics, excessive growth of ROS in microalgae cells breaks the dynamic balance. The metabolism of organic xenobiotics (including pharmaceutical pollutants) in plants is similar to that in animals, including the functionalization phase, conjugation phase, and compartmentalization phase. ROS originating from the functionalization phase includes a set of enzymatic transformations (including cytochrome P450 monooxygenase, peroxidases, and peroxygenases) or is derived from electron transport systems in the chloroplast [31,32,33]. The antioxidant system is one of the most critical mechanisms of algal cell response to antibiotic stress [9]. The oxidative stress and the antioxidants of nonenzyme and enzymes induced by SMX, ERY, and their mixture in *R. subcapitata* showed different trends (Figure 3). As a biomarker of lipid peroxidation in algae cells, the increased malondialdehyde (MDA) contents in the *R. subcapitata* after exposure to high concentrations of SMX, ERY, and their mixture suggested induced cellular lipid peroxidation (Figure 3a). The stimulatory effects on proliferation stem from a certain rise in ROS, while strong inhibition is ascribed to a decline in intracellular ROS [34]. The inhibition mechanism of *R. subcapitata* exposure to SMX at a low concentration (0.3 mg/L) was different from other tests. Excessively low levels of ROS cannot sustain normal signal transfer, which may cause the growth of *R. subcapitata* to be inhibited [35,36]. 

In this study, for *R. subcapitata* exposed to SMX, SOD activity significantly (*p* < 0.01) increased by 44% and 56% at 0.5 and 0.7 mg/L SMX concentrations, and SOD activity insignificantly decreased at ≤0.3 mg/L SMX concentrations (Figure 3b). The decrease in SOD activity may be caused by the high accumulation of H_2_O_2_. The imbalance between the detoxification rate and H_2_O_2_ production leads to the accumulation of H_2_O_2_, further inhibiting SOD activity in cells. Reduced SOD activity will cause the further accumulation of H_2_O_2_ [37]. When *R. subcapitata* was exposed to ERY and the mixture, SOD activity increased significantly at concentrations relatively higher than those studied (Figure 3b). Earlier studies showed that enhanced SOD activity is a crucial mechanism for plants to resist organic pollution [15]. It was reported that the SOD activity of *C. mexicana* increased significantly when exposed to CIP at 60 and 100 mg/L [15]. Increased SOD activity facilitates the removal of excess ROS [38].

CAT is an important enzyme promoting the conversion of H_2_O_2_ to ground-state oxygen and H_2_O. Normally, CAT is used to treat high concentrations of H_2_O_2_, and the concentration of H_2_O_2_ affects the activity of CAT [39]. GSH-Px can catalytically reduce the conversion of GSH to oxidized glutathione disulfide (GSSG) and convert H_2_O_2_ to H_2_O, thereby protecting cell membrane structure and function. In the present work, exposure to SMX (0.5 and 0.7 mg/L) and the mixture (0.077 mg/L) resulted in a significant increase in the CAT activity of *R. subcapitata* relative to the control group. Nevertheless, the opposite result was found with exposure to ERY (Figure 3c). GSH and GST are the two enzymes critical in the removal of ROS and protect plants from exogenous chemicals. GST act as a peroxidase and directly detoxifies xenobiotics with electrophilic groups by conjugating with GSH [40,41]. In the present study, the high concentrations of SMX and the mixture caused GSH and GST activity to significantly decrease (Figure 3d,e). The content of GSH may also be the reason for the difference in GST activity between the three test conditions, as GSH can act as an activator of GST and SOD in plants [42]. GSH is considered to be the substrate required for GSH-Px and GST enzymes to decompose hydroperoxides [43]. The decrease in GSH activity indicates that SMX has a certain inhibitory effect on the synthesis of GSH. It was reported that GSH synthesis is closely related to photosynthesis, but SMX is a photosynthesis inhibitor that can cause damage to photosynthesis systems and lead to a decrease in the amount of GSH synthesis [44]. The effects of the mixture on the GSH content of *R. subcapitata* may follow a similar pattern. When *R. subcapitata* were exposed to SMX, ERY, and their mixture, GSH-Px activity had no significant change (Figure 3f). GSH-Px were activated unsuccessfully and/or reduced change under SMX so that the alga is not fully competent to remove H_2_O_2_ [41]. As described above, the removal capacity of H_2_O_2_ affects the activity of SOD. 

### 2.4. pH Value Change

**Figure 4:**
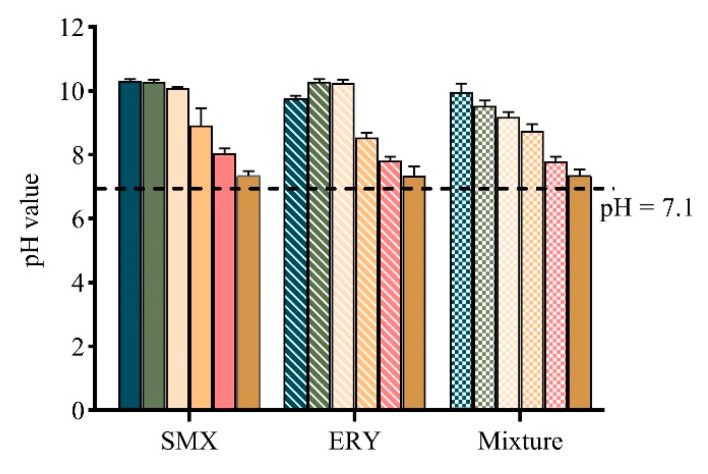
The pH value of *R. subcapitata* after 11 days exposure to SMX, ERY, and their mixture. SMX (0, 0.1, 0.3, 0.5, 0.7 and 0.9 mg/L), ERY (0, 0.01, 0.03, 0.05, 0.07, and 0.09 mg/L), and their mixture (0, 0.011, 0.033, 0.055, 0.077 and 0.099 mg/L). Data are presented as mean values ± standard deviation (n = 3).

After 11 days of exposure, the pH values in the medium of all tests were increased (Figure 4). This is probably due to the fact that the carbon source for algal growth is from the conversion of bicarbonates to CO_2_ [45]. An increase in the pH values of the medium can enhance the ionization of the acidic antibiotics and lower their toxicity. Therefore, the toxicity of SMX (pKa, 5.7) may be lower than the initial state during cultivation, whereas for ERY (pKa, 8.89), it is not [43,46]. The lipophilicity of a compound is most commonly expressed as the octanol/water partition coefficient (Kow), and the higher the log Kow value, the higher the lipophilicity. In the present study, ERY had a greater effect on the microalgae than SMX, which may be because of the high lipophilic degree of ERY (log Kow, 3.06) compared to SMX (log Kow, 0.89), resulting in a strong interaction between algae and the pharmaceuticals and the severe impairment of biochemical parameters of the cells [27,43,47].

## 3. Materials and Methods

### 3.1. Test Algae and Culture

*Raphidocelis subcapitata* (*R. subcapitata*, FACHB-271) is a planktonic species that lives in global freshwater lakes and rivers. *R. subcapitata* as the model algae species was widely used in the toxicity test. The algae were cultivated in Erlenmeyer flasks containing 150 mL of Blue–Green (BG11) Medium (adjust pH to 7.1 with 1 M NaOH or HCl). The alga was cultured within a constant temperature incubator (at 22 ± 2 °C under 75 μmol photons m^−2^ s^−1^). During the culture, all flasks were shaken three times a day to accelerate cell dispersion in the culture medium. The placement location of each Erlenmeyer flask was stochastically changed to make sure the illumination intensity of each group was kept as accordant as possible to reduce accidental errors. All flasks were capped with perforated transparent plastic film and autoclaved at 121 °C for 30 min. The test algae were precultured for 14 days to foster an optimal growth state. 

### 3.2. Antibiotics

Sulfamethoxazole (SMX) and erythromycin (ERY) belong to different kinds of antibiotics (sulfonamides and macrolide, respectively) and are generally detected in surface water. The action model of antibiotics to small aquatic plants may be similar to bacteria. Therefore, SMX and ERY may cause adverse effects on algae growth. SMX (≥98.0% purity) and ERY (≥98.0% purity) were dissolved in methanol and then diluted with BG11 medium as a stock solution. The final contents of methanol were less than 0.01% (*v*/*v*), which reduced experimental error. The diluted SMX and ERY were added to the medium for single and mixed exposure tests. All other chemicals used were of analytical grade. 

### 3.3. Procedures for the Growth Inhibition and Ecotoxicological Risk Assessment

The toxicity test was undertaken following OECD 201. All glassware used in the tests was autoclaved at 121 °C for 30 min before use. The antibiotics were filtered with a 0.22 μm sterilized syringe filter. The toxicology tests of SMX (0, 0.1, 0.3, 0.5, 0.7, and 0.9 mg/L), ERY (0, 0.01, 0.03, 0.05, 0.07, and 0.09 mg/L), and their mixture (0, 0.011, 0.033, 0.055, 0.077, and 0.099 mg/L) on *R. subcapitata* were investigated. The ratio of SMX/ERY (10:1) was chosen on the basis of their half-maximal effective concentration (EC_50_). Toxicant concentrations presented in this work are in the form of nominal concentration. The microalgal suspension (at exponential phase) was diluted to 150 mL in 250 mL Erlenmeyer flasks using BG11 with antibiotics. The growth of *R. subcapitata* was determined by a hemocytometer to calculate the change of algae cells at regular time intervals (2, 4, 7, and 11 days) during the cultivation. The concentration of the test samples was determined by chemical analysis. All the experiments were conducted in triplicates. 

The ecotoxicological risk assessment of pollutants can be predicted by risk quotients (RQs). RQs, defined as the measured environmental concentration (MECs) divided by the predicted no-effect concentration (PNEC), are commonly used in risk assessments [48]. The RQs were calculated using Equation (1):

RQ = MEC/PNEC(1)

where MEC used to calculate the RQ is founded on the maximum environmental concentrations of pollutants in surface waters and wastewater reported in previous studies. PNEC is the EC_50_ value divided by an assessment factor of 1000 [49].

### 3.4. Photosynthetic Pigment Content

A 3 mL volume of each culture was filtered through a 0.22 μm filter at regular time intervals. The filter was placed in a centrifuge tube containing 3 mL of methanol and stored in an ultra-low-temperature freezer to extract photosynthetic pigment content. After 24 h, it was harvested by centrifugation at 10,000 rpm for 10 min. The absorbance of the supernatant at 666, 653, and 470 nm was measured by a microplate reader. Each experimental measurement was corrected for algae wet weight. The contents of chlorophyll a, b, and total carotenoids were calculated using the following equations [49]:

Chlorophyll a (mg/L) =15.65 A_666_ − 7.34 A_653_ (2)

Chlorophyll b (mg/L) =27.05 A_653_ − 11.21 A_666_ (3)

Total carotenoids (mg/L) = (1000 A_470_ − 44.76 A_666_)/221 (4)

where A666, A653, and A470 are the absorption value at wavelengths of 666, 653, and 470 nm, respectively.

### 3.5. Analysis of Antioxidant Responses

In the process of the experiment, a 3 mL algae culture medium was extracted from each Erlenmeyer flask to measure oxidative stress biomarkers at 11 days. The samples needed to be pretreated before the measurement of oxidative stress biomarkers, and the culture medium was centrifuged at 4000 rpm for 10 min. The supernatant was discarded. The cell pellet was resuspended in 1 mL of normal saline to wash, centrifuged again at 1000 rpm for 10 min, and the supernatant was discarded. The wash operation was repeated three times. A 2 mL volume of normal saline was added to the cell pellet then mixed well and homogenized manually, 1 min at a time at 30 s intervals, and repeated 6–8 times. Oxidative stress biomarkers were measured using a Tecan Infinite^®^ 200 Pro Multifunction microplate reader (Tecan Austria GmbH, Grödig, Austria). Measurement of oxidative stress biomarkers was strictly performed according to the manufacturer’s instructions.

### 3.6. Antibiotic Analyses

In this study, the initial concentrations of SMX and ERY were confirmed by liquid chromatography–tandem mass spectrometry (LC-MS/MS, Agilent, Santa Clara, CA, USA) analysis coupled with a C18 column (ZORBAX Eclipse Plus column 600 bar, 3 mm × 100 mm × 1.8 μm). Details of the sample pretreatment, instrumental setting, and method validation are listed in the Supplementary Materials (Supplementary Tables S1–S3). 

### 3.7. Statistics Analysis

All the experiments were conducted in triplicate. Dunnett’s multiple comparisons test (one-way analysis of variance) was used to statistically analyze the data using GraphPad Prism version 8.0.2 software for Windows (USA). Values were considered significant when *p* < 0.05. 

## 4. Conclusions

The toxicity of SMX, ERY, and their mixture was investigated depending on the growth inhibition of *R. subcapitata*, which suggested that SMX was more toxic than ERY. In this experiment, the RQ values of SMX, ERY, and their mixture were significantly greater than one, indicating high potential risks of these antibiotics. *R. subcapitata* took different strategies to eliminate or alleviate the oxidative damage caused by ROS. *R. subcapitata* can reduce the SMX-induced and mixture-induced ROS by the increased activities of SOD and CAT and reduce the ERY-induced ROS by the increased activities of SOD, GSH, and GST. Moreover, high concentrations of antibiotics lead to a decrease in chlorophyll a and total carotenoid content, affecting the ability to photosynthesize ROS scavenging capacity. This may be a factor leading to the inhibition of algal growth. There were some differences in the contents of nonenzymatic antioxidants and enzyme antioxidants in *R. subcapitata*, indicating that the antioxidant mechanisms of the *R. subcapitata* were not identical exposure to different antibiotics. All in all, our work is mainly to study the toxicity of different antibiotics to microalgae and the antioxidant mechanism of microalgae treatment of ROS.

## Figures and Tables

**Table 1:** Effective concentration (EC_50_) and risk quotients of SMX, SMZ, and their mixture (SMX: ERY = 10:1) for *R. subcapitata.*

Time (Days)	2	4	7	11
EC_50_ of SMX (mg/L)	0.76	0.49	1.27	1.55
RQ of SMX surface water	5.35 ^a^	8.31 ^a^	3.20 ^a^	2.63 ^a^
RQ of SMX wastewater	15.66 ^a^	23.22 ^a^	9.37 ^a^	7.68 ^a^
EC_50_ of ERY (mg/L)	0.069	0.044	0.046	0.082
RQ of ERY surface water	5.07 ^b^	7.95 ^b^	7.61 ^b^	4.27 ^b^
RQ of ERY wastewater	12.02 ^b^	18.86 ^b^	18.24 ^b^	10.12 ^b^
EC_50_ of mixture (mg/L)	0.16	0.06	0.08	0.07
RQ of mixture surface water	27.63 ^c^	73.67 ^c^	55.25 ^c^	63.14 ^c^
RQ of mixture wastewater	79.44 ^c^	211.83 ^c^	158.88 ^c^	181.57 ^c^

^a^ The maximum concentration of SMX used to calculate the RQ of surface water and wastewater in Table 1 was 4.07 and 11.9 μg/L, respectively [12,13]. ^b^ The maximum concentration of ERY used to calculate the RQ of surface water and wastewater in Table 1 was 0.35 and 0.81 μg/L, respectively [13,14]. ^c^ The maximum concentration of the mixture used to calculate the RQ of surface water and wastewater in Table 1 was 4.42 and 12.71 μg/L, respectively [12,13,14].

**Table 2:** Summary of ecotoxicological effects of SMX and ERY on microalgal species in terms of growth inhibition, obtained in this study and compared with previously reported work.

Algae species	EC_50_ of SMX (mg/L)	Major Focus of the Study	References
*Chlorella vulgaris*	48 h EC_50_ = 1.51 96 h EC_50_ = 0.98	Ecotoxicological evaluation	[17]
*Synechococcus leopolensis Raphidocelis subcapitata* *Raphidocelis subcapitata*	96 h EC_50_ = 0.0268 96 h EC_50_ = 0.146 72 h EC_50_ = 0.52	Ecotoxicological evaluation Ecotoxicological evaluation Ecotoxicological evaluation	[18,19]
*Scenedesmus obliquus*	96 h EC_50_ = 0.018	Ecotoxicology evaluation, modeling of toxicity, risk assessment	[5]
Algae species *Raphidocelis subcapitata* *Dunaliella tertiolecta* *Raphidocelis subcapitata*	EC_50_ of ERY (mg/L) 72 h EC_50_ = 0.02 96 h EC_50_ = 0.0272 h EC_50_ = 0.38	Ecotoxicological evaluation Ecotoxicological evaluation	[20,21]

## Data Availability

The data presented in this study are available on request from the corresponding author. The data are not publicly available due to the project being not complete.

## Supplementary Materials

The following are available online at https://www.mdpi.com/article/10.3390/antibiotics10050576/s1. Table S1: HPLC program on the condition of positive electrospray ionization; Table S2: Optimized retention time, ion transitions, ion transitions, collision cell exit potential for MS/MS determination of target antibiotic; Table S3: Actual concentration of SMX and ERY in algal medium (mg/L).
