# Supplementary material for: Combined Effects of Sulfamethoxazole and Erythromycin on a Freshwater Microalga, Raphidocelis subcapitata: Toxicity and Oxidative Stress
Source: Antibiotics (Basel). 2021 May 13;10(5):576. doi: 10.3390/antibiotics10050576 (PMC8153177; doi:10.3390/antibiotics10050576)
Supplement: Supplementary file 1 [file antibiotics-10-00576-s001.zip › antibiotics-1208155-supplementary.pdf]

Supplementary material for

Combined effects of sulfamethoxazole and erythromycin on a freshwater microalga, *Raphidocelis subcapitata*: toxicity and oxidative stress

Yibo Zhang [1], Da He [2], Fang Chang [3], Chenyuan Dang [1,*], Jie Fu [1]

[1] School of Environmental Science and Engineering, Huazhong University of Science and Technology, Wuhan 430074, China

[2] Key Laboratory of Ecological Impacts of Hydraulic Projects and Restoration of Aquatic Ecosystem of Ministry of Water Resources, Institute of Hydroecology, Ministry of Water Resources & Chinese Academy of Sciences, Wuhan, China.

[3] Marine Resources Research Centre, Tianjin Research Institute for Water Transport Engineering, M.O.T., Tianjin 300456, China

SPE methods and processes

The collected samples were diluted to 50 mL with culture medium, and each sample was spiked with 30 ng of erythromycin-13C-d3 and Sulfamethazine-d4, as the internal surrogate standard. Samples were extracted by pressurized liquid extraction using solid phase extraction unit (Shanghai Nai Precision Instrument Co., Ltd.) Placed the Oasis® HLB solid phase extraction cartridge (3cc, 60 mg, Waters, USA) on the extraction unit and then activated with 3 mL of methanol and 3 mL of ultrapure water. The sample solution was passed through the cartridge at flow rate of 5–10 mL/min, rinsed the beakers by 10 mL ultra-pure water after samples were all passed through the cartridges, and then evacuated for one hour. After that, the analyte was eluted into the test tube four times with methanol, 3 mL each time. Under a 37°C water bath, dry under a nitrogen stream. The samples of different concentration were reconstituted with Different amounts of methanol. Then Added different volume of Atrazine-d5 internal standards (IS) to these groups, and made the concentration of Atrazine-d5 in each sample is 20 ng/mL. Mixed well and filtered with 0.22 μm micron nylon membrane. The sample solution was then transferred to an autosampler vial (with insert) for LC/MS/MS analysis.

Supplementary Table 1. HPLC program on the condition of positive electrospray ionization.

**Column**	**Agilent ZORBAX Eclipse Plus C18 HPLC column (3×100 mm, 1.8 μm)**		
Mobile phase	A: ultrapure water with 0.1% formic acid (v/v)		
	B: Acetonitrile		
Column Temperature	24°C		
Injection volume	10 μL		
Flow rate	0.3 mL/min		
Gradient	Time (min)	A (%)	B1(%)
	0.00	92.5	7.5
	1.00	92.5	7.5
	3.00	88.0	12.0
	4.50	80.0	20.0
	6.00	40.0	60.0
	9.00	10.0	90.0

	10.00	10.0	7.5
	11.00	92.5	7.5

Supplementary Table 2. Optimized retention time, ion transitions, ion transitions, collision cell exit potential for MS/MS determination of target antibiotic.

Compound	Retention time (min)	Ion transitions (m/z)	Collision energy (eV)	Collision cell exit potential (V)
Sulfamethoxazole	7.06	254 > 155.9	80	15
		254 > 108		25
Sulfamethazine-d4	7.073	283.1 > 124.2	124	30
		283.1 > 186.1		20
Atrazine-d5	8.579	221.0 > 101.0	113	30
		221.0 > 137.0		25
		221.0 > 179.0		20
Erythromycin	7.400	734.5 > 158.2	140	30
		734.5 > 576.4		20
Erythromycin-13C-d3	7.151	738.6 > 162.0	146	34
		738.6 > 582.5		19

Supplementary Table 3. Actual concentration of SMX and ERY in algal medium (mg/L).

SMX		ERY		Mixture (SMX: ERY=10:1)	
Nominal concentration	Actual concentration	Nominal concentration	Actual concentration	Nominal concentration	Nominal concentration
0	0.001	0	0	0	0
0.1	0.098	0.01	0.011	0.01+0.001	0.011+0.0012
0.3	0.292	0.03	0.027	0.03+0.003	0.029+0.0029
0.5	0.511	0.05	0.049	0.05+0.005	0.049+0.0052
0.7	0.681	0.07	0.072	0.07+0.007	0.069+0.0071
0.9	0.934	0.09	0.091	0.09+0.009	0.088+0.0101
